# Topical Application of *Chrysanthemum indicum L.* Attenuates the Development of Atopic Dermatitis-Like Skin Lesions by Suppressing Serum IgE Levels, IFN-**γ**, and IL-4 in Nc/Nga Mice

**DOI:** 10.1155/2012/821967

**Published:** 2012-02-09

**Authors:** Sunmin Park, Jung Bok Lee, Suna Kang

**Affiliations:** ^1^Department of Food and Nutrition, Hoseo University, 165 Sechul-Ri, BaeBang-Yup, Asan-Si, ChungNam-Do 336-795, Republic of Korea; ^2^Department of Mechatronics Engineering, Hoseo University, Asan 336-795, Republic of Korea

## Abstract

*Chrysanthemum indicum L*. (CIL) is widely used as an anti-inflammatory agent in Asia and our preliminary study revealed that CIL reduced interleukin (IL)-4 and IL-13 in 2,4-dinitrochlorobenzene (DNCB)-treated HaCaT cells, a human keratinocyte cell line. We investigated the atopic dermatitis (AD) effect of topically applied CIL in mice with AD-like symptoms. After topical application of 1,3-butylen glycol (control), CIL-Low (5%), CIL-High (30%), or 0.1% hydrocortisone (HC) on the AD-like skin lesions in DNCB-treated NC/Nga mice for 5 weeks, the ear thickness, mast cell infiltration, and serum immunoglobulin E (IgE), IgG1, IL-4 and interferon (IFN)-**γ** were measured. The gene expressions of IL-4, IL-13, and IFN-**γ** in the dorsal skin were assayed. CIL treatment dosedependently reduced severity of clinical symptoms of dorsal skin, ear thickness, and the number of mast cells and eosinophils. CIL-High significantly decreased serum IgE, IgG1, IL-4, and IFN-**γ** levels and reduced mRNA levels of IFN-**γ**, IL-4, and IL-13 in dorsal skin lesion. The improvement by CIL-High was similar to HC, but without its adverse effects such as skin atrophy maceration, and secondary infection. In conclusion, CIL may be an effective alternative substance for the management of AD.

## 1. Introduction

Atopic dermatitis (AD) is a common skin disease characterized by chronic and relapsing inflammatory dermatitis with immunological disturbances [1]. The incidence of AD is continuously increasing worldwide with a prevalence rate of approximately 10–20% and is more common among infants and children. Most patients with AD have increased circulating eosinophils and immunoglobulin (Ig) E due to elevated interleukin (IL)-4, IL-5, and IL-13 produced by T-helper (Th) 2 cells [2, 3]. Th2 immune responses are known to play an important role in the pathogenic mechanism of AD. In addition, Th1 immune response related to interferon-gamma (IFN-*γ*) is also associated with the pathophysiology of AD. Therefore, AD is known to be a biphasic inflammatory skin disease, provoked by Th1/Th2 immune responses [4]. Acute AD skin lesions exhibit Th2 type inflammatory cytokine profiles, whereas the chronic phase is characterized by Th1 type immune responses releasing Th1 cytokines such as IFN-*γ* and IL-12 and delayed-type hypersensitivity reactions [4, 5]. The imbalance of Th1 and Th2 immune responses plays important roles in the development of AD [6, 7].

NC/Nga mice are the most extensively studied animal model of AD since the inbred strain was established in 1957 [8]. These mice spontaneously develop AD-like eczematous skin lesions when kept in air-uncontrolled conventional housing, but not when maintained under specific pathogen-free (SPF) conditions [8]. However, when these mice are maintained under conventional conditions, the low incidence of AD-like lesions is a drawback [8]. In contrast, chemical hapten, 2,4-dinitrofluorobenzene (DNFB), or 2,4-dinitrochlorobenzene (DNCB) application produces 100% reproducible AD-like lesions in NC/Nga mice [8–10]. Both DNCB- and DNFB-induced contact hypersensitivity is a T-cell-mediated inflammatory skin reaction that is believed to be associated with Th1 activation [11, 12]. Since the clinical symptoms displayed in the NC/Nga mice treated with chemicals and humans with AD are similar, the mice are considered to be AD animal model suitable for understanding and evaluating effective therapeutic strategies for AD [8]. Since topical hydrocortisone exerts anti-inflammatory, antipruritic, and vasoconstrictive actions, it is widely accepted that topical hydrocortisone therapy is crucial for the management of AD. However, it cannot be used for long periods, and adverse side effects are frequently observed like burning, itching, irritation, dryness, folliculitis, hypertrichosis, acneiform eruptions, hypopigmentation, perioral dermatitis, allergic contact dermatitis, maceration of the skin, secondary infection, skin atrophy, and striae.

Accordingly, a wide variety of natural products are currently being evaluated for alternative AD therapies, with the ideal agent possessing potent efficacy with a minimal side effect profile [10, 13, 14]. *Chrysanthemum indicum L*. is widely used for anti-inflammatory agents in Southeast Asian folk medicine. It was also found to have anti-microbial, anti-oxidant, and anti-inflammatory action [15, 16]. *Chrysanthemum indicum L*. inhibits inflammatory mediators, such as NO, PGE_2_, TNF-*α*, and IL-1*β*, via suppression of MAPKs and NF-*κ*B-dependent pathways [15]. In addition, our preliminary study revealed that the 1,3-butylene glycol extract of dried flowers of *Chrysanthemum indicum L*. decreases the expression of IL-4 and IL-13 the most in DNCB-treated HaCaT cells, human keratinocytes, when the extracts of 23 herbs including *Chrysanthemum indicum L*. were compared. These effects may be due to the sesquiterpene compounds such as kikkanol A, B, and C and flavone glycones such as eriodictyol 7-O-*β*-D-glucopyranosiduronic acid, acaciin, and luteolin 7-O-*β*-D-glucopyranoside [17, 18]. Thus, *Chrysanthemum indicum L*. may relieve AD-like symptoms. However, it has not yet been determined whether *Chrysanthemum indicum L*. suppresses the development and progression of AD. In this study, we used the DNCB-treated NC/Nga murine model to examine the inhibitory effect of *Chrysanthemum indicum L*. on the development and progression of AD-like skin lesions.

## 2. Materials and Method

### 2.1. Preparation of *Chrysanthemum indicum L.* Extracts

Dried flowers of *Chrysanthemum indicum L.* were purchased in Kyung-Dong Herb market (Seoul, Korea) in 2008, identified by Dr. Joo YS (Department of Herbology, Woosuk University, Wanju-gun, Korea), and a voucher specimen (No. 2008-05 and 2008-06) deposited at the herbarium of Department of Food & Nutrition, Hoseo University. Since 1,3-butylene glycol is a good solvent for making skin lotion, dried flowers of *Chrysanthemum indicum L.* (1 kg) were extracted at room temperature for 12 hours with 20 or 3.3 L of 1,3-butylene glycol, filtered, and the filtrates centrifuged at 450 ×g to make 5 or 30% extracts. When more than 30% *Chrysanthemum indicum L.* was extracted with 1,3-butylene glycol, the extract formed a precipitate. The supernatants were used for topical application.

### 2.2. Animals

Twenty female 6-week-old NC/Nga mice were purchased from Charles River Japan (Yokohama, Japan) and maintained under conventional conditions: a 12 h light/12 h dark cycle, room temperature of 22-23°C, and humidity of 55 ± 15%. The mice had free access to food and water. All surgical and experimental procedures were performed according to the guidelines of the Animal Care and Use Review Committee at Hoseo University, Korea.

### 2.3. Induction of AD-Like Skin Lesion

Mice were anesthetized with a mixture of ketamine and xylazine (100 and 10 mg/kg body weight), after which the animal's back hair and right ear were shaved 1 day prior to sensitization. In the first day, 1% DNCB in acetone/olive oil (3 : 1) was applied to the dorsal skin and right ear then 0.2% DNCB was applied every other day [19]. The same volume of acetone/olive oil vehicle was applied instead of DNCB solution to controls.

### 2.4. Topical Application of 1,3-Butylene Glycol Extracts of *Chrysanthemum indicum L.*

To determine the AD effect of* Chrysanthemum indicum L.*, two dosages were assigned based on the preliminary cell-based study and the maximum dosage to extract. A preliminary study showed that 50 *μ*g/mL of *Chrysanthemum indicum L. *was effective against an AD model in HaCaT cells, a human keratinocyte cell line, but not 5 *μ*g/mL. After the induction of the AD-like skin lesions, animals were divided into four groups of 10 mice each. These groups were then treated topically in the dorsal skin and right ear for 5 weeks with one of four agents: 1,3-butylene glycol (BG; control), 5 or 30% dried flower of *Chrysanthemum indicum L.* (CIL-Low or CIL-High), or 0.1% hydrocortisone butyrate (HC; positive control) twice a day. Mice with no induction of AD-like skin lesion were treated with 1,3-butylene glycol as a normal control. 

### 2.5. Evaluation of Skin Lesion Severity

The relative dermatitis severity was assessed macroscopically using the following scoring procedure. The total skin severity score was defined as the sum of the individual scores for each of the following four signs: (1) erythema and hemorrhage, (2) edema, (3) erosion (excoriation), and (4) scaling (dryness) [20]. In this system, 0 was defined as exhibiting no symptoms, 1 as mild symptoms, 2 as moderate symptoms, and 3 as severe symptoms. Additionally, the mice were photographed once per week.

Ear thickness was measured before and after induction of the inflammatory response using a digital micrometer (MSI-Viking, Duncan, SC). The micrometer was applied near the tip of the ear just distal to the cartilaginous ridges, and the thickness was recorded in micrometers. To minimize technique variations, a single investigator performed the measurements throughout each experiment.

### 2.6. Measurement of Serum IgG1 and IgE Levels and Iterleukin-4 (IL-4) and Interferon-*γ* (IFN-*γ*) Cytokine Levels

The total serum IgG1 and IgE was quantified by sandwich enzyme-linked immunosorbent assay (ELISA) Quantitation Kit (R & D Systems, Minneapolis, MN, USA) according to the manufacturer's protocol. The serum concentrations of the cytokines (IL-4 and IFN-*γ*) were also quantified using a mouse cytokine enzyme immunoassay kit (R & D Systems).

### 2.7. Real-Time Quantitative Reverse Transcriptase-Polymerase Chain Reaction (RT-PCR)

The dorsal skin tissues from five mice from each group were collected at the end of treatment. Total RNA was isolated from the skin tissues using a monophasic solution of phenol and guanidine isothiocyanate (Trizol reagent, Gibco-BRL, Rockville, MD), followed by extraction and precipitation with isopropyl alcohol. The cDNA was synthesized from equal amounts of total RNA with superscript III reverse transcriptase, and polymerase chain reaction (PCR) was performed with high-fidelity Taq DNA polymerase. Equal amounts of cDNA were mixed with SYBR Green supermix (Bio-Rad) mix and were analyzed using a realtime PCR machine (BioRad Laboratories, Hercules, CA). The expression level of the gene of interest was corrected for that of the house keeping gene, *β*-actin. The following primers were used for PCR reactions (5′-3′), mouse IFN-*γ*, sense 5′-CGGCACAGTCATTGAAAGCCTA-3′, and antisense 5′-GTTGCTGATGGCCTGATTGTC-3′; IL-4, sense 5′-TCTCGAATGTACCAGGAGCCATATC-3′, and antisense 5′-AGCACCTTGGAAGCCCTACAGA-3′; mouse IL-13, sense 5′-cagctccctggttctctcac-3′, and antisense 5′-ccacactccataccatgctg-3′; mouse *β*-actin, sense 5′-CATCCGTAAAGACCTCTATGCCAAC-3′, and antisense 5′-ATGGAGCCACCGATCCACA-3′. The primers were designed to sandwich at least one intron in order to distinguish between the products derived from mRNA and genomic DNA.

### 2.8. Histological Analysis

Dorsal samples were taken 24 h after final DNCB administration on day 35 and they were fixed in 10% buffered neutral formaldehyde and embedded in paraffin wax. Histological sections were 6 *μ*m thick and were stained with haematoxylin and eosin for counting number of eosinophils. The sections were also stained with 0.5% toluidine blue for investigating the number of mast cells. The cell counts were performed in six consecutive microscopic fields at 400x magnification. 

### 2.9. Statistical Analysis

Statistical analysis was performed using SAS software and all results expressed as mean ± standard deviation. The biological and metabolic effects of CIL-Low, CIL-High, HC (positive control), and vehicle (a negative control) were compared by one-way ANOVA. Significant differences in the main effects among the groups were identified by Tukey's test at *P* < 0.05. The significance of differences between the mice with and without AD-like skin lesion was determined by two-sample *t* test.

## 3. Results

### 3.1. Severity of Skin Lesion

**Figure 1:**
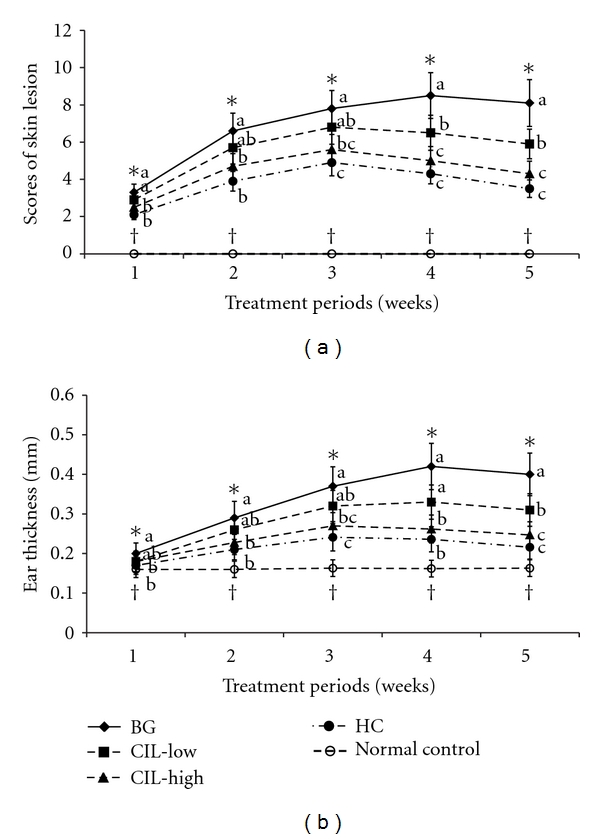
Changes in severity scores and ear thickness of atopic dermatitis (AD) in NC/Nga mice. AD was induced in Nc/Nga mice by topical application of 2,4-dinitrochlorobenzene (DNCB) in the dorsal skin, and a right ear was topically treated with 1,3-butylen glycol (BG; control), 5% Chrysanthemum indicum L. (CIL-Low), 30% Chrysanthemum indicum L. (CIL-High), and 0.1% hydrocortisone (HC) on the lesions for 5 weeks. Normal controls did not have DCNB-induced AD. CIL decreased clinical severity of atopic dermatitis symptoms and ear thickness in a dose-dependent manner, and CIL-High had a statistically similar effect on the severity as HC. (a) Changes in severity scores. (b) Changes of ear thickness. Each value represents the mean ± SD of 10 mice in each group. *Significantly different among the different treatments in Nc/Nga mice at *P* < 0.05. a, b, c Values with different superscripts were significantly different among Nc/Nga mice by Tukey test at *P* < 0.05. ^†^Significantly different between control (BG) and normal control at *P* < 0.05.

The NC/Nga mice developed AD-like skin lesions following repeated application of DNCB as evidenced by skin lesion scores, whereas normal controls did not exhibit any skin lesions. The total scores were calculated from sums of the scores for erythema, edema, erosion, and dryness; with a score of 12 indicating the most severe state. The normal controls exhibited no changes in skin lesion scores over time (Figure 1(a)). In accordance with the previous finding, the clinical severity of skin lesions in the control group increased gradually depending on the times of challenge with DNCB. All mice in the control group treated with BG (control) exhibited AD-like skin lesions including erythema, edema, erosion, and dryness in the dorsal skin (Figure 1(a)). However, topical application of CIL dose-dependently lowered the skin lesion scores below that of the controls: CIL-Low significantly decreased the clinical scores at 4 and 5 weeks from the DNCB sensitization in comparison to the control, and CIL-High lowered the scores more than CIL-Low (Figure 1(a)). The decreased clinical scores in CIL-High were similar to those observed with HC treatment. These results indicated that CIL-Low and CIL-High reduced the progression of AD-like skin lesions in comparison to the control, and CIL-High exhibited the similar improvement to HC. The scores increased after topical application and began to be lowered from day 21 in CIL-Low and CIL-High, whereas they reached a maximum at day 28 in the BG group (Figure 1(a)). 

### 3.2. Ear Thickness

Ear thickness gradually increased after topical application of DNCB in BG and CIL-Low groups up to the 4th week but in CIL-High and HC groups, only up to the 3rd week (Figure 1(b)). This indicated that the AD symptoms were alleviated after the peak of ear thickness. Ear thickness in the CIL-Low group significantly lowered only at the 4th week while CIL-High suppressed its increase in comparison to the BG group from the 2nd week of sensitization (Figure 1(b)). Ear thickness of the CIL-Low and CIL-High groups was significantly different at the 4th and 5th weeks. The decrease in the CIL-High group was not significantly different from HC (Figure 1(b)). Ear thickness did not change in the normal control group during the experimental periods. 

### 3.3. Serum IgG1, IgE, IL-4, and INF-*γ* Levels

**Figure 2:**
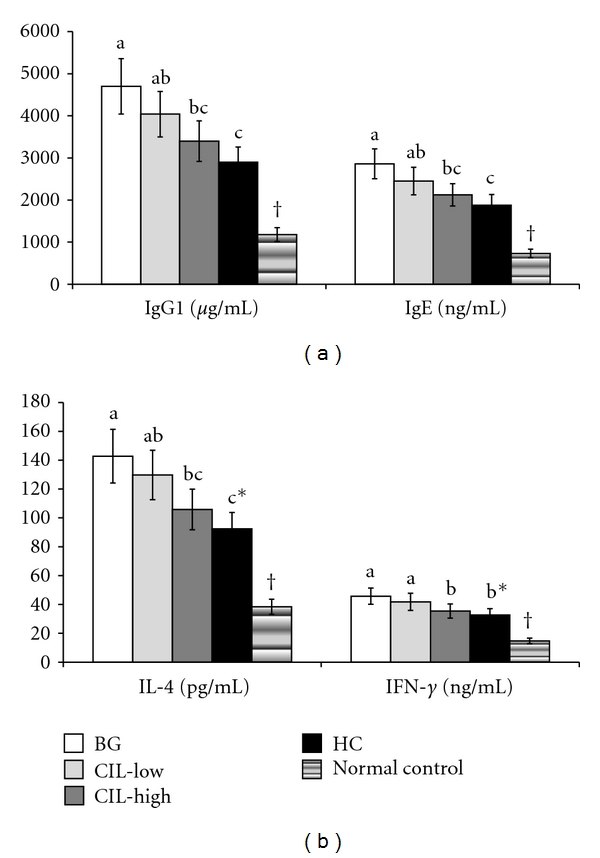
Serum levels of IgG1, IgE, IL-4, and IFN-*γ* levels at the end of experimental periods. AD was induced in Nc/Nga mice by topical application of 2,4-dinitrochlorobenzene (DNCB) in the dorsal skin, and a right ear was topically treated with 1,3-butylen glycol (BG; control), 5% *Chrysanthemum indicum L.* (CIL-Low), 30% *Chrysanthemum indicum L.* (CIL-High), and 0.1% hydrocortisone (HC) on the lesions for 5 weeks. Normal controls did not have DCNB-induced AD. After 5 weeks of treatments, serum was separated to measure immunoglobulins and cytokines. CIL-High significantly reduced circulating levels of cytokines and immunoglobulins in comparison to the control, and CIL-High exhibited a statistically similar amelioration to HC. (a) Serum levels of IgG1 and IgE. (b) Serum levels of IL-4 and INF-*γ*. Each value represents the mean ± SD of 10 mice in each group. *Significantly different among the different treatments in Nc/Nga mice at *P* < 0.05. a, b, c Means on the bars with different superscripts were significantly different among Nc/Nga mice by Tukey test at *P* < 0.05. ^†^Significantly different between control (BG) and normal control at *P* < 0.05.

At day 35 after the sensitization, serum IgG1 and IgE levels were higher in the control group than in the normal control group, but the levels were dosedependently reduced by CIL treatments in DNCB-treated mice (Figure 2(a)). CIL-Low and CIL-High decreased circulating levels of IgG1 and IgE, but it was statistically significant only for CIL-High which had levels close to those of HC. IL-4 produced by Th2 cells was much greater in the control group than the normal control group whereas only CIL-High significantly suppressed the increase in DNCB-treated mice, and the suppression was as much as in HC, a positive control (Figure 2(b)). The control group also exhibited higher levels of INF-*γ* produced by Th1 cells than the normal control group, but CIL-High lowered the levels in DNCB-treated mice, but it did not reach levels of the normal control group (Figure 2(b)). The reduction of serum INF-*γ* levels was statistically similar to HC. The ratio of IL-4 and IFN-*γ* was higher in control group than normal control, whereas the ratio was dosedependently lowered by CIL in DNCB-treated mice, but only CIL-High significantly decreased the ratio. The ratio was statistically similar between CIL-High and HC (data not shown).

### 3.4. Histological Findings and Mast Cell Counts in the Inflamed Skin

**Figure 3:**
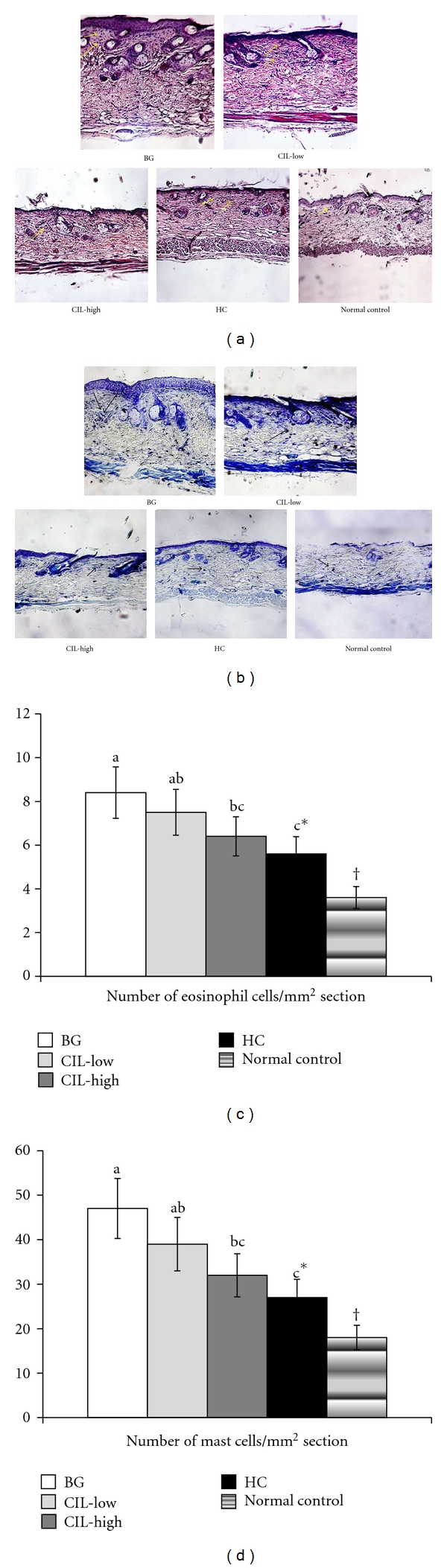
The number of mast cells and eosinophils of the dorsal skin with histopathological analysis. AD induced in Nc/Nga mice by topical application of 2,4-dinitrochlorobenzene (DNCB) in the dorsal skin, and a right ear was topically treated with 1,3-butylen glycol (BG; control), 5% *Chrysanthemum indicum L.* (CIL-Low), 30% *Chrysanthemum indicum L.* (CIL-High), and 0.1% hydrocortisone (HC) on the lesions for 5 weeks. Normal controls did not have DCNB-induced AD. After 5 weeks of treatments, the dorsal skin was fixed with 10% formaldehyde, embedded in paraffin, and then sections were made. The skin sections were stained with hematoxylin and eosin and toluidine blue staining. CIL-High significantly elevated mast cells and eosinophils in the lesion of the dorsal skin in comparison to the control and exhibited a statistically similar increment to HC. Each value represents the mean ± SD of 5 mice in each group. (a) The number of eosinophils as determined by hematoxylin and eosin staining. Eosinophils exhibited as blue dots. (b) The number of mast cells determined by toluidine blue staining. Mast cells appear as blue dots. *Significantly different among the different treatments in Nc/Nga mice at *P* < 0.05. a, b, c Means on the bars with different superscripts were significantly different by Tukey test at *P* < 0.05. ^†^Significantly different between control (BG) and normal control at *P* < 0.05.

The dorsal skin of the mice in the control groups exhibited hypertrophy, hyperkeratosis, intercellular edema, the liquefaction degeneration of the basal layer, and infiltration of inflammatory cells such as mast cells and eosinophils in contrast to the normal control group (Figure 3). The symptoms seen in dorsal skin such as hypertrophy, hyperkeratosis, intercellular edema, and liquefaction degeneration of the basal layer were relieved by the treatments with CIL in a dose-dependent manner. As seen in Figure 3(a), hypertrophy, hyperkeratosis, intercellular edema, and liquefaction degeneration of the basal layer were ameliorated with CIL-Low and CIL-High treatment in comparison to the control group (Figure 3(a)). The improvement of the symptoms in CIL-High was similar to the improvements in the HC group.

An increased number of mast cells and eosinophils were observed in the skin lesions of the control group compared to the normal control group (Figures 3(a) and 3(b)). The increase in mast cell and eosinophil counts was dose dependently suppressed by CIL treatment in DNCB-treated mice. CIL-Low decreased the mast cells and eosinophils in comparison to the control, but it was not significantly different. CIL-High significantly lowered them, and the decrease was similar to the HC group (Figures 3(a) and 3(b)).

### 3.5. Cytokine mRNA Expression Levels

**Figure 4:**
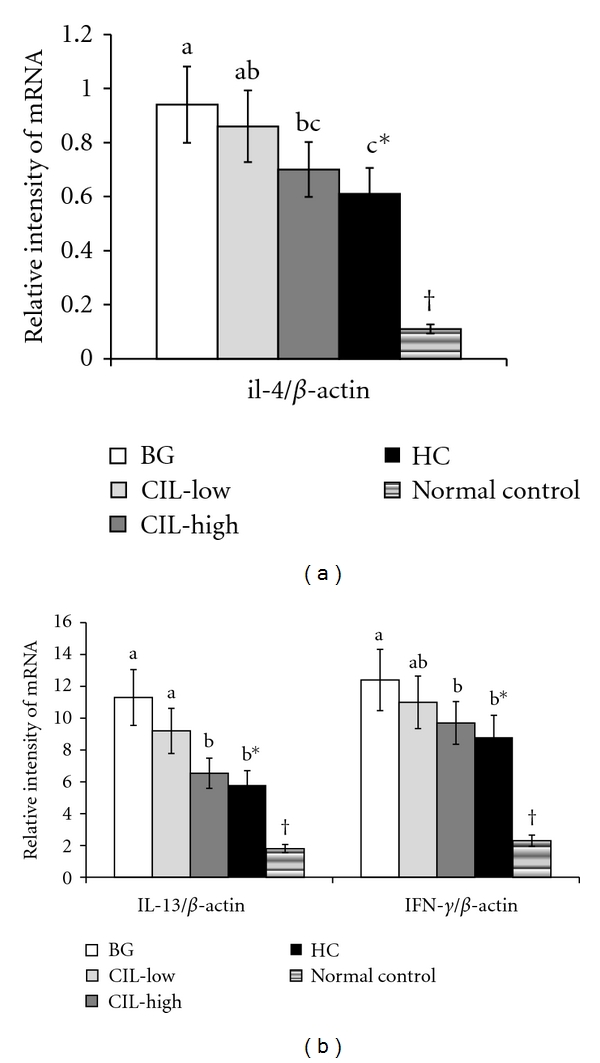
IL-4, IL-13, and IFN-*γ* expression in the dorsal skin. AD induced in Nc/Nga mice by topical application of 2,4-dinitrochlorobenzene (DNCB) in the dorsal skin, and a right ear was topically treated with 1,3-butylen glycol (BG; control), 5% *Chrysanthemum indicum L.* (CIL-Low), 30% *Chrysanthemum indicum L.* (CIL-High), and 0.1% hydrocortisone (HC) on the lesions for 5 weeks. Normal controls did not have DCNB-induced AD. After 5 weeks of treatments, total RNA was extracted from the dorsal skin, and cDNA was generated. The mRNA expression of IL-4, IL-13, and INF-*γ* was measured by realtime PCR, and their relative expression was standardized according to respective *β*-actin mRNA levels. CIL-High significantly decreased the expression of IL-4, IL-13, and INF-*γ* in the lesion of the dorsal skin in comparison to the control and exhibited a statistically similar decrease as HC. Each value represents the mean ± SD of 5 mice in each group. *Significantly different among the different treatments in Nc/Nga mice at *P* < 0.05. a, b, c Means on the bars with different superscripts were significantly different by Tukey test at *P* < 0.05. ^†^Significantly different between control (BG) and normal control at *P* < 0.05.

To determine cytokine production in the inflamed dorsal skin lesions, mRNA expression of IL-4, IL-13, and INF-*γ* cytokines was analyzed. The dorsal skin tissues of DNCB-treated control mice exhibited much higher expression levels of IL-4, IL-13, and IFN-*γ* than those of the normal controls (Figure 4). CIL suppressed the mRNA expression of IL-4 and IL-13 produced by Th2 cells in a dose-dependent manner, while the expression of IFN-*γ* produced by Th1 was also reduced by CIL treatment (Figure 4). However, the decreases in mRNA levels were significantly different only in CIL-High, not in CIL-Low, and the reduction of INF-*γ* was less than that of IL-4 and IL-13 in CIL treatment. The decreased expressions of IL-4, IL-13, and IFN-*γ* by CIL-High were similar to those following treatment with HC, a positive control (Figure 4).

## 4. Discussion

Atopic dermatitis is a biphasic inflammatory skin disease, provoked by an imbalance between Th1 and Th2 immune responses [4]. Th2 immune responses are mediated by interleukin (IL)-4, IL-5, and IL-13 while Th1 immune responses are modulated by IFN-*γ* [2]. In particular, Th2 responses are key elements to the pathogenesis of atopic disorders. NC/Nga mice were the first mouse model of AD reported by Matsuda et al. [21], and the mice treated with DFBN, DNCB, or picryl chloride have also been used as an animal model for human AD [22]. Elevated levels of serum total IgE have been reported to correlate with the appearance of the AD-like lesions in NC/Nga mice, with massive infiltration of IL-4- and IL-13-producing Th2 cells and the degranulation of mast cells and eosinophils [4]. HC is a potent topical corticosteroid used to alleviate rash, eczema, and dermatitis. However, HC is a steroidal agent with adverse effects such as facial hypertrichosis, folliculitis, miliaria, and genital ulcers [23], which has inspired to investigations of nonsteroidal agents for relieving AD. Recently, several studies have reported that Herbal Oriental Medicine therapy may be effective in AD patients [24]. The present study showed that CIL dose dependently reduced the severity of AD-like skin lesions by decreasing serum IgE and IgG1 levels and infiltration of mast cells and eosinophils in DNCB-treated NC/Nga mice. The results also indicate that the effects of CIL were probably due to a decrease in IFN-*γ*, IL-4, and IL-13 production by activated Th1 and Th2 cells. CIL-High was found to be as effective as HC for alleviating AD.

Mast cells are known as key effector cells in IgE-mediated allergic disorders and are activated by cross-linking of a high affinity IgE receptor [3]. Upon activation, mast cells undergo degranulation and release a variety of biologically active substances, which play an important role in host defense and allergic reactions including AD. Infiltration of mast cells into the dermis is a necessary characteristic for defining an appropriate animal model for AD [25]. We investigated the efficacy of CIL for preventing AD-like skin lesions in DNCB challenged NC/Nga mice and its mechanism for preventing and alleviating AD was explored since CIL has been widely used as an anti-inflammatory agent in Southeast Asian folk medicine. We found that CIL-High significantly suppressed the numbers of mast cells infiltrating in the skin lesions of the atopic dermatitis mice, suggesting that the activation and migration of mast cells may be an immunopharmacological target of CIL-High.

It is known that mast cell activation is tightly modulated by IgE from B cells, and increased total serum IgE levels are a hallmark of AD [24]. AD induced by DNFB application activates B cells in NC/Nga mice by a Th2 reaction through IL-4 that elevates IgE production [25]. IgE expression is known to cause both acute and chronic phase skin symptoms. Consistent with these reports, we found that serum IgE levels were significantly increased by repeated DNCB application in NC/Nga mice, as was IL-4 and IL-13 production by activated Th2 cells. These cytokines are known to play important roles in the inflammation and hypertrophy of the skin in AD [25]. We also found that higher IgE, IL-4, and IL-13 levels were associated with more severe skin lesions. Topical application of CIL-High significantly improved the severity of AD-like skin lesions by suppressing serum IgE, IL-4, and IL-13 levels. The improvement by CIL-High was as effective as with HC.

In addition to cytokines released from Th2 cells, Th1 cytokines such as INF-*γ* are involved in AD development [4]. Under normal conditions, the differentiation of naive T cells to Th1 and Th2 lineages is regulated by cytokines that are secreted from various cells, including themselves, and the Th1/Th2 balance is maintained [6, 7]. However, in atopic dermatitis, the balance shifts to Th2 dominance; this eventually leads to excessive Th2 cytokine production [6]. Some studies have found that increased INF-*γ* levels alleviate AD-like symptoms, but those results remain controversial. In the present study, repeated DNCB application increased both IFN-*γ* and IL-4 production from activated CD4^+^ T cells of draining lymph nodes in NC/Nga mice. CIL dose dependently decreased both Th1 (INF-*γ*) and Th2 cytokines (IL-4 and IL-13) as effectively as HC. Furthermore, the ratio of Th1 to Th2 cytokines was increased by CIL treatment.

In conclusion, CIL-High reduced the development of AD-like skin lesions resulting from repeated DNCB application in NC/Nga mice by suppressing total serum IgE levels and IFN-*γ* and IL-4 production by activated CD4^+^ T cells. CIL-High ameliorated AD symptoms as effectively as HC. Sesquiterpenes and flavonoids in CIL may be the active components exerting anti-AD-like effects; further study is needed to identify the primary active components.
